# Overexpression of *Jatropha curcas* *ERFVII2* Transcription Factor Confers Low Oxygen Tolerance in Transgenic Arabidopsis by Modulating Expression of Metabolic Enzymes and Multiple Stress-Responsive Genes

**DOI:** 10.3390/plants9091068

**Published:** 2020-08-20

**Authors:** Piyada Juntawong, Pimprapai Butsayawarapat, Pattralak Songserm, Ratchaneeporn Pimjan, Supachai Vuttipongchaikij

**Affiliations:** 1 Department of Genetics, Faculty of Science, Kasetsart University, Bangkok 10900, Thailand; pimprapai.bu@ku.th (P.B.); p.songserm017480@gmail.com (P.S.); ratchaneeporn.pj@gmail.com (R.P.); fsciscv@ku.ac.th (S.V.); 2 Center for Advanced Studies in Tropical Natural Resources, National Research University-Kasetsart University, Bangkok 10900, Thailand; 3 Omics Center for Agriculture, Bioresources, Food and Health, Kasetsart University (OmiKU), Bangkok 10900, Thailand

**Keywords:** abiotic stress, RNA-seq, transcription factor, waterlogging

## Abstract

Enhancing crop tolerance to waterlogging is critical for improving food and biofuel security. In waterlogged soils, roots are exposed to a low oxygen environment. The group VII ethylene response factors (ERFVII*s*) were recently identified as key regulators of plant low oxygen response. Oxygen-dependent N-end rule pathways can regulate the stability of ERFVIIs. This study aims to characterize the function of the *Jatropha curcas ERFVIIs* and the impact of N-terminal modification that stabilized the protein toward low oxygen response. This study revealed that all three JcERFVII proteins are substrates of the N-end rule pathway. Overexpression of *JcERFVII2* conferred tolerance to low oxygen stress in Arabidopsis. In contrast, the constitutive overexpression of stabilized *JcERFVII2* reduced low oxygen tolerance. RNA-seq was performed to elucidate the functional roles of *JcERFVII2* and the impact of its N-terminal modification. Overexpression of both wildtype and stabilized *JcERFVII2* constitutively upregulated the plant core hypoxia-responsive genes. Besides, overexpression of the stabilized *JcERFVII2* further upregulated various genes controlling fermentative metabolic processes, oxidative stress, and pathogen responses under aerobic conditions. In summary, JcERFVII2 is an N-end rule regulated waterlogging-responsive transcription factor that modulates the expression of multiple stress-responsive genes; therefore, it is a potential candidate for molecular breeding of multiple stress-tolerant crops.

## 1. Introduction

Waterlogging can damage most crops, creating one of the most significant problems in agriculture worldwide. During the heavy rainy season in the plain area, soil can quickly become waterlogged due to poor drainage, creating a low oxygen environment in the root area underground. Low oxygen stress leads to the induction of a particular set of genes involved in carbohydrate utilization, energy metabolism, and fermentation to sustain ATP production [1]. Over the long term, low oxygen stress means morphological adaptation is required to keep the level of oxygen under control [2]. Since global climate change could increase the number of flooding events, improved crop varieties with waterlogging tolerance are essential [3,4].

The ethylene response factor (*ERF*) family is one of the largest plant-specific transcription factor families characterized by a single DNA-binding domain, APETALA2 (AP2), with expanded functions in hormonal response, development, and tolerance to biotic and abiotic stresses [5,6,7]. Group VII ERF (ERFVII) transcription factors is a subgroup of ERF*s* that has been recognized as a critical factor controlling the expression of numerous genes involved in an adaptive response to low oxygen stress in model plants [8,9,10,11]. A characteristic feature of all Arabidopsis *ERFVIIs* (*RAP2.2*, *RAP2.3*, *RAP2.12, HRE1*, and *HRE2*) is a conserved N-terminal motif (N-degron; [5,12]), which enables them to be degraded by oxygen and nitric oxide (NO)-dependent N-end rule pathways [13,14,15]. Overexpression of the Arabidopsis *ERFVIIs* enhances flooding or low oxygen stress tolerance in transgenic Arabidopsis plants [13,14,16,17,18,19,20,21]. Interestingly, overexpression of the stabilized (N-terminal mutation) *HRE1* and *HRE2* in Arabidopsis further improved low oxygen tolerance compared to Arabidopsis lines overexpressing the wildtype *HRE1* and *HRE2* [14]. However, enhanced stability of *RAP2.12* resulted in reduced biomass under aerobic conditions and did not increase tolerance to low oxygen stress in transgenic Arabidopsis plants [13,22]. Transcription of genes encoding for fermentative and starch degradation enzymes were constitutively activated in transgenic Arabidopsis overexpressing stable *RAP2.12*, which negatively affected growth and development under the aerobic condition and reduced tolerance to low oxygen stress [22]. In rice, previous studies identified *ERFVIIs*, *Snorkels*, and *Sub1A*, as a key player orchestrating the escape and quiescence response needed to survive flash-flood and prolonged submergence, respectively [23,24]. Although Sub1A contained an N-degron, it was not a substrate of the N-end rule pathway [11,14]. It has recently been shown that *Sub1A* transcriptionally activates the other *ERFVII*s, *ERF66*, and *ERF67*, resulting in transcriptional accumulation of anaerobic survival genes and improved submergence tolerance in rice [11]. Remarkably, constitutive expression of the stabilized wheat *ERFVII*, *TaERFVII.1*, enhanced tolerance to waterlogging in transgenic wheat without negative impacts on development and grain yield under aerobic conditions [25]. Thus, identification, selection, and modification of the *ERFVII* genes could be a valuable approach to improve crop waterlogging tolerance.

*Jatropha curcas* is a drought-tolerant oilseed crop for biodiesel production. It can be grown on marginal land without competing with other food crops [26]. However, waterlogging caused a significant reduction of growth and biomass yield, suggesting that Jatropha is extremely sensitive to waterlogging [27,28]. Undoubtedly, genetic improvement of waterlogging tolerant Jatropha is needed to increase Jatropha oil production. Previously, we transcriptionally profiled gene expression in Jatropha and found that waterlogging promoted anaerobic respiration, but inhibited carbohydrate synthesis, cell wall biogenesis, and plant growth [29]. Based on our previous study, *ERFVIIs* had been proposed as candidate genes for genetic engineering of waterlogging tolerant Jatropha [29].

In this study, we cloned and evaluated the tissue-specific expression and waterlogging responsive pattern of Jatropha *ERFVIIs* (*JcERFVIIs*). Next, we followed up by examining the N-end rule regulated protein stability of *JcERFVIIs* and overexpressing the *JcERFVII2* genes in Arabidopsis to evaluate the flooding and low oxygen tolerant phenotype. Finally, the molecular function of *JcERFVII2* was further investigated by transcriptome profiling of transgenic Arabidopsis lines.

## 2. Results

### 2.1. Cloning and Bioinformatics Analysis of JcERFVII Genes

**Figure 1:**
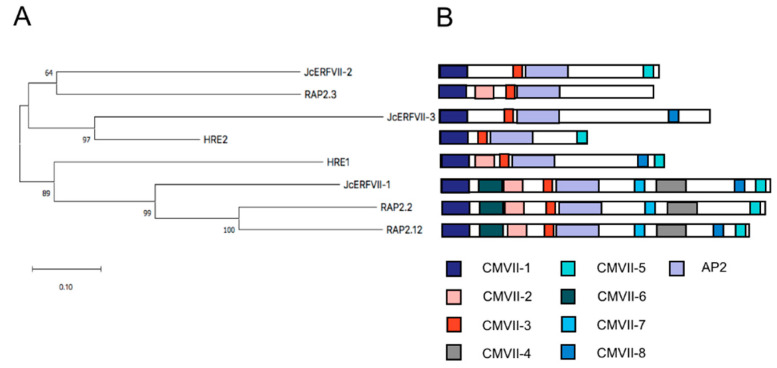
Phylogenetic and domain architecture analysis of Jatropha ERFVIIs. (**A**) The phylogenetic tree based on the amino acid sequence of Arabidopsis and Jatropha ERFVIIs. The numbers are bootstrap values after 1000 replicates. Scale bar represents genetic distance. (**B**) Diagram representing domain architecture of Jatropha ERFVIIs following previously published motifs [5].

Previously, we identified three *JcERFVIIs*, namely *JcERVII1* (*Jcr4S00420.40*), *JcERFVII2* (*Jcr4S00982.160*), and *JcERFVII3* (*Jcr4S01651.60*), from the Jatropha genome [29]. All three *JcERFVIIs* possess a conserved N-degron signal [NH_2_-MCGGAII(A/S)D] [29]. The full-length open reading frames (ORFs) of *JcERFVIIs* were cloned. Sequence analysis reveals that *JcERFVII1*, *JcERFVII2*, and *JcERFVII3* are composed of 1158, 762, and 945 nucleotides, respectively (Supplementary Materials Data S1). Deduced amino acid sequences of *JcERFVII1*, *JcERFVII2*, and *JcERFVII3* provided encoded proteins with 385, 253, and 314 amino acids with predicted molecular weights of 43, 29, and 36 kD, respectively. The amino acid sequences of the *JcERFVIIs* were aligned with amino acid sequences from all five members of the *Arabidopsis ERFVIIs*, including *RAP2.2*, *RAP2.3*, *RAP2.12*, *HRE1*, and *HRE2*, and the phylogenetic relationship was evaluated. The results revealed that *JcERFVII1* clustered with *RAP2.2* and *RAP2.12*, *JcERFVII2* clustered with *RAP2.3*, and *JcERFVII3* clustered with *HRE2* (Figure 1A). Based on the previously reported Arabidopsis ERFVII protein domain data [5], MEME assisted domain analysis also showed the similarity among each phylogenetic cluster (Figure 1B).

### 2.2. Tissue-Specific and Waterlogging Expression Patterns of JcERFVIIs

**Figure 2:**
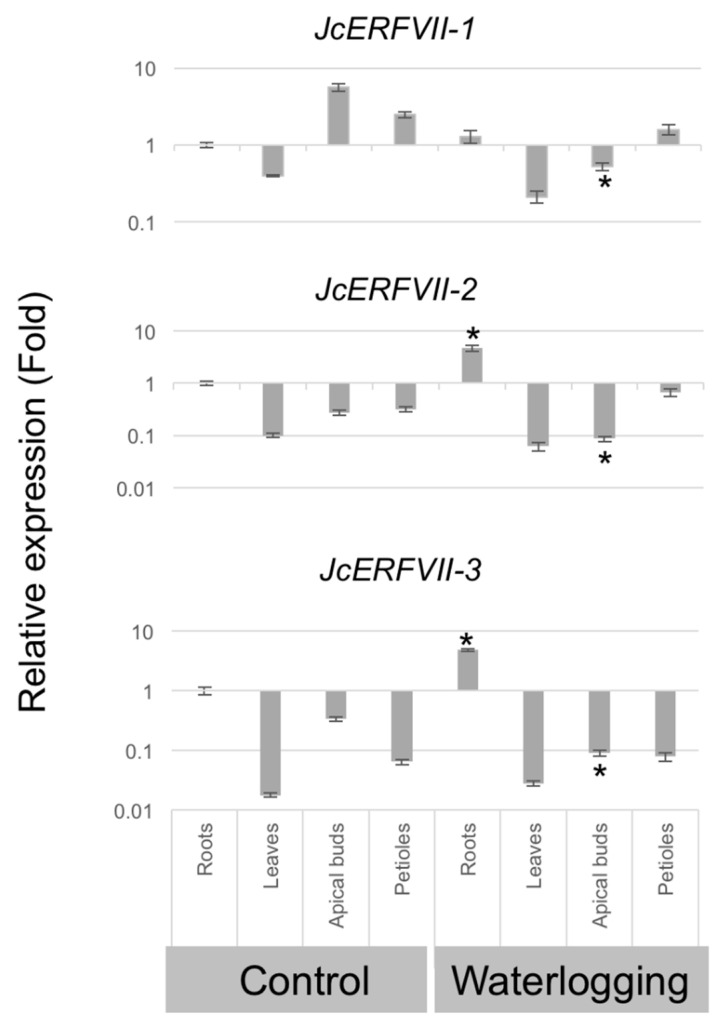
Quantitative analysis of *JcERFVII* expression. Relative expression values of *JcERFVIIs 1–3* from roots, leaves, apical buds, and petioles of Jatropha seedlings under control and 1 d waterlogging. Relative expression was normalized to the abundance of *UBQ10*. Data represent mean ± SE (*n* = 3). Asterisks indicate *p* < 0.05 (*t*-test).

We examined the expression pattern of the three *JcERFVIIs* in the tissue of Jatropha seedlings using qRT-PCR (Figure 2). Under aerobic conditions, the expression of all three *JcERFVIIs* can be found in roots, leaves, apical buds, and petioles of Jatropha seedlings. *JcERFVII1* exhibited the highest expression in apical buds and the lowest expression in leaves, while *JcERFVII2* and *JcERVII3* exhibited the highest expression in roots and the lowest expression in leaves. We also compared the expression levels of the three *JcERFVIIs* using the transcriptome data from roots, leaves, stems, and shoot apexes collected in a publically available *J. curcas* database (JCDB) [30]. We found that among the three *JcERFVIIs*, *JcERFVII1,* and *JcERFVII3* displayed the highest and the lowest expression, respectively (Supplementary Materials Figure S1). Moreover, the expression levels of *JcERFVII2* in Jatropha tissues were more uniform than those of the others (Supplementary Materials Figure S1).

To explore whether the *JcERFVIIs* are related to waterlogging response, we examined the expression patterns of *JcERFVIIs* in Jatropha seedlings subjected to 24 h soil waterlogging. In the waterlogged root, the expression of *JcERFVII2* and *JcERFVII3* was significantly increased, while the expression of *JcERFVII1* remained unaffected (Figure 2). Besides, waterlogging resulted in a significant reduction of *JcERFVII1*, *JcERFVII2*, and *JcERFVII3* expression in apical buds (Figure 2).

### 2.3. Stability of JcERFVIIs In Vitro

**Figure 3:**
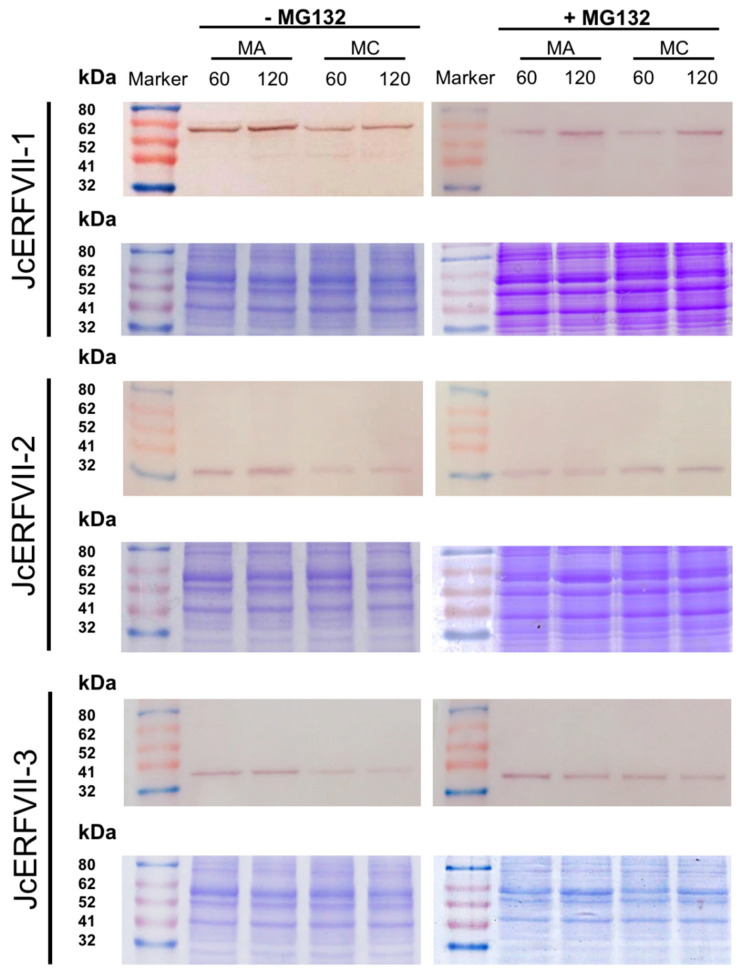
Jatropha ERFVIIs are substrates for the N-end rule pathway in vitro. Western blot analysis of in vitro stability of HA-tagged wildtype (MC) and stable mutation (MA) of JcERFVIIs 1–3 in the absence or presence of proteasome inhibitor (MG132). 60 and 120 indicate incubation time in minutes. Coomassie staining of a similar SDS-PAGE used for western blotting was used as a loading control.

Since all three JcERFVII proteins possess a conserved N-degron, we hypothesized that they are targets of the N-end rule pathway. We used a previously established in vitro assay by which proteins are expressed in a rabbit reticulocyte system containing essential components for the N-end rule pathway [14]. Western blot analysis of in vitro translated JcERFVII proteins tagged with a haemagglutinin (3xHA) epitope demonstrated a single band with the migration pattern corresponding to their predicted molecular weight (Figure 3). Our results demonstrated that mutation of cysteine to alanine at amino acid residue position 2 (MA) in all three JcERFVIIs increased protein stability after 60 and 120 min incubation periods (Figure 3). We also showed that supplementation of MG132, a proteasome inhibitor, increased the accumulation of wildtype (MC) JcERFVII proteins in vitro (Figure 3). These data strongly suggest that JcERFVIIs are substrates of the N-end rule pathway.

### 2.4. Overexpression of the JcERFVII2 Enhanced Low Oxygen Tolerance in Arabidopsis

Based on previous studies, among the five members of the Arabidopsis *ERFVIIs*, the role of *RAP2.3* in low oxygen responses has been less explored. Therefore, we aim to characterize *JcERFVII2* function towards low oxygen response. To investigate the function of *JcERFVII2* in providing tolerance to low oxygen stress and whether modulation of its stability could affect the stress tolerance, we generated transgenic Arabidopsis lines overexpressing *MA-* or *MC-JcERFVII2* driven by the CaMV35S promoter. Ten and 5 transgenic lines overexpressing *MA-* and *MC-JcERFVII2* were generated and 4 independent homozygous lines, 3*5S:MA-JcERFVII2-1* (MA-Line1), *35S:MA-JcERFVII2-7* (MA-Line7), *35S:MC-JcERFVII2-3* (MC-Line3), and *35S:MC-JcERFVII2-5* (MC-Line5), were selected for functional analysis. Semi-quantitative RT-PCR analysis confirmed the expression of *JcERFVII-2* in the transgenic lines (Supplementary Materials Figure S2A).

**Figure 4:**
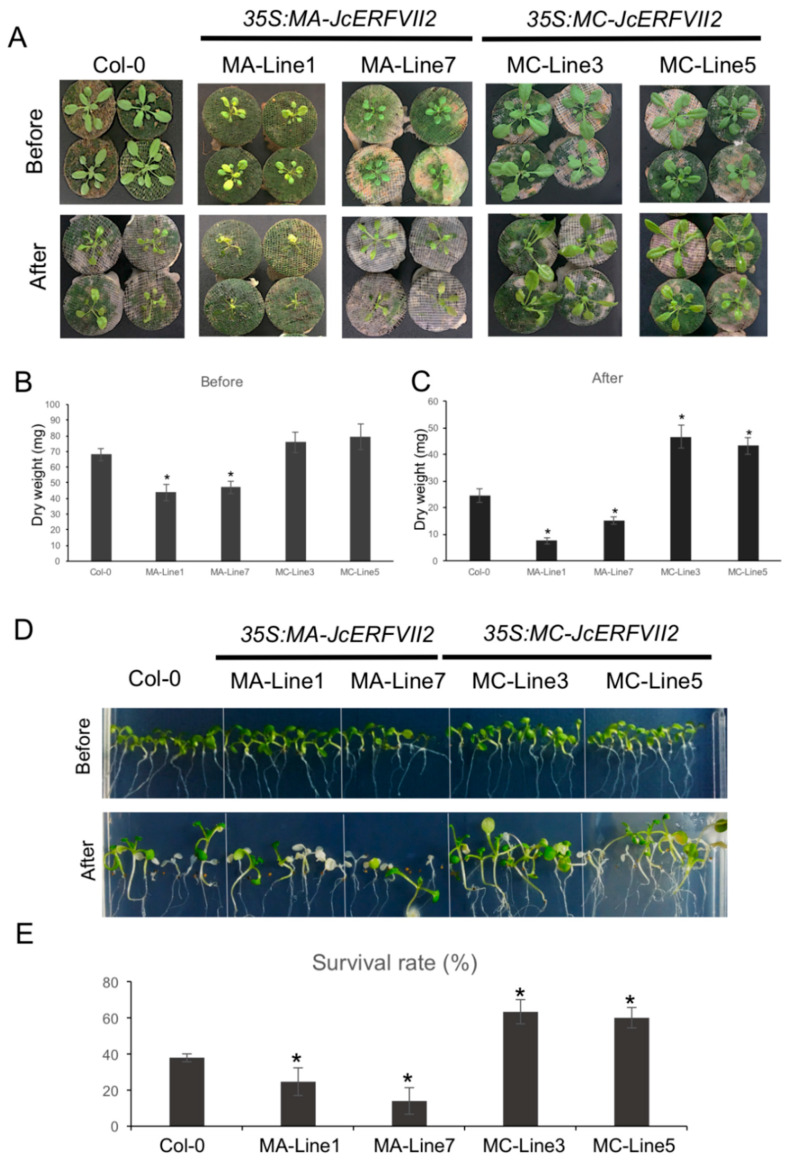
Overexpression of wildtype *JcERFVII2* confer low-oxygen tolerance. (**A**) Phenotype of wildtype Arabidopsis (Col-0) Arabidopsis transgenic lines overexpressing wildtype (MC) and stable (MA) *JcERFVII2* subjected to 3 d submergence stress. (**B**) Dry weight of 3-week-old rosette leaves before submergence (*n* = 10). (**C**) Dry weight of rosette leaves after 3 d submergence (*n* = 10). (**D**) Phenotypes of the Arabidopsis transgenic seedlings overexpressing *MA-* and *MC-JcERFVII2* after 3 d hypoxia and 3 d recovery. (**E**) Percentage of seedling survival for wildtype Arabidopsis (Col-0) and the Arabidopsis lines overexpressing *MA* and *MC-JcERFVII2*. Data are means of triplicate experiments. Each experiment contains 6–10 plants/genotype. Error bars represent SD. Asterisks indicate *p* < 0.05 (*t*-test).

For submergence stress, the four transgenic lines and the wildtype *A. thaliana* Col-0 were grown until reaching the 10 leaf-stage and subjected to submergence stress for 3 d (Figure 4A). While overexpression of *MC-JcERFVII2* did not show any effect on the phenotype of the transgenic lines (MC-Line3 and MC-Line5) (Figure 4A,B), it considerably improved submergence tolerance with respect to the wildtype, as demonstrated by the increases of dry weight after submergence (Figure 4C). On the other hand, transgenic lines overexpressing *MA-JcERFVII2* (MA-Line1 and MA-Line7) showed reduced plant growth when grown under aerobic conditions (Figure 4A,B) and decreased submergence tolerance when compared with the wildtype (Figure 4A,C).

For low oxygen survival assay, after 3 d of 2% oxygen and 3 d of recovery under aerobic condition, MC-Line3 and MC-Line5 showed significantly higher survival rate (63% and 60%, respectively) than that of the wildtype (38%) (Figure 4D,E). However, MA-Line1 and MA-Line7 displayed a significantly lower survival rate (24% and 13%, respectively) (Figure 4D,E). Together, these results clearly demonstrated that the constitutive overexpression of *MC-JcERFVII2* could enhance growth and survival under low oxygen in transgenic Arabidopsis, while that of *MA-JcERFVII2* resulted in growth reduction under aerobic conditions and poorly performed under low-oxygen stress.

### 2.5. Transcriptome Profiling of Transgenic Arabidopsis Overexpressing JcERFVII2

**Figure 5:**
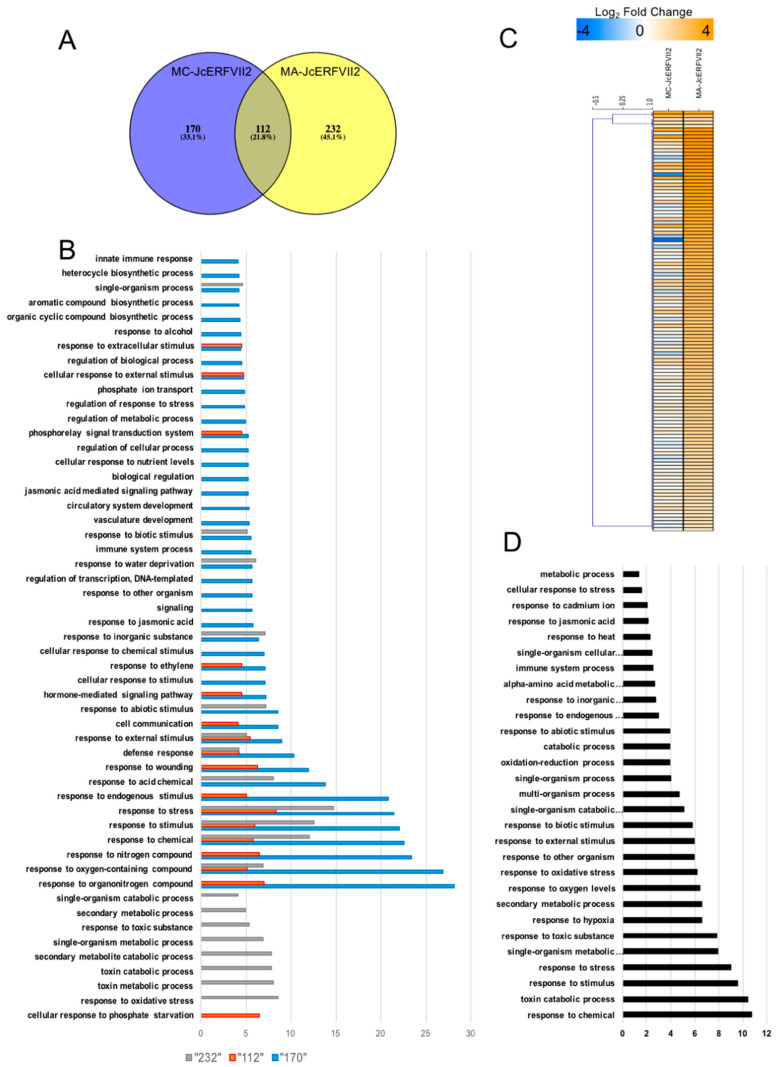
Overexpression of *JcERFVII2* upregulates multiple stress-responsive genes in Arabidopsis. (**A**) Venn diagram of differentially expressed genes (DEGs) from the Arabidopsis lines overexpressing *MA-* and *MC-JcERFVII2*. (**B**) Enrichment of GO terms from DEGs of the Arabidopsis lines overexpressing *MA-* and *MC-JcERFVII2*. Bar chart represents –log10 adjusted *p*-values of enrichments GO terms. (**C**) Heat map represents the expression pattern of the upregulated DEGs derived from the Arabidopsis lines overexpressing *MA-* and *MC-JcERFVII2*. (**D**) Selected enrichment GO terms of the upregulated DEGs found in (**C**). Black bar represents –log10 adjusted *p*-values of enrichments GO terms. Data used to generate this figure can be found in Supplementary Materials Tables S1 and S2.

To analysis the impact of the N-terminal modification on the molecular function of the *JcERFVII2* gene, we profiled the transcriptome of transgenic Arabidopsis overexpressing *MA-* and *MC-JcERFVII2* (MA-Line1 and MC-Line3, respectively) using Col-0 as a control genotype. Two biological replicates of total RNAs from 7 d.o. seedlings grown in aerobic conditions were isolated and subjected to RNA-seq. RNA-seq reads were mapped to the *A. thaliana* TAIR10 genome. The number of reads aligned back to each gene was obtained for differential gene expression analysis. Transcriptome analysis identified 344 and 282 differentially expressed genes (DEGs) with significant changes in gene expression as evaluated by false discovery rate (FDR) < 0.05 from *MA-* or *MC-JcERFVII2* overexpressing lines, respectively (Figure 5A; Supplementary Materials Table S1). Of 282 DEGs from the MC-Line3, 29 DEGs (10%) were upregulated, and 253 DEGs (90%) were downregulated (Supplementary Materials Table S1), while, of 344 DEGs in the MA-Line1, 122 DEGs (35%) were upregulated, and 222 DEGs (65%) were downregulated (Supplementary Materials Table S1). Venn’s diagram analysis revealed that 112 DEGs were commonly found in both *MA-* and *MC-JcERFVII2* transgenic lines, while 232 DEGs and 170 DEGs were exclusively found in *MA-* and *MC-JcERFVII2* transgenic lines, respectively (Figure 5A). It should be noted that the endogenous ERFVIIs were not differentially expressed in transgenic lines overexpressing both *MA-* and *MC-JcERFVII2* (Supplementary Materials Table S1). To confirm that, we obtained the CPM (count per million) expression values from our RNA-seq data. Mostly, the expression of the endogenous ERFVII genes in transgenic lines is similar to the Col-0 (Supplementary Materials Figure S2B).

Gene ontology (GO) analysis was performed to obtain the overview of *JcERFVII2* regulated genes using an FDR cutoff of <1.00 × 10^−4^ (Figure 5B). The results demonstrated that *JcERFVII2* regulated genes function in cellular metabolic processes and several aspects of stress responses, as observed in the enriched GO terms derived from both *MA-* and *MC-JcERFVII2* DEGs (Figure 5B). GO terms related to response to stress, stimulus, chemical, and oxygen-containing compounds were enriched in the 112, 170, and 232 DEGs previously described (Figure 5B). Interestingly, 8 out of 49 core hypoxia-responsive genes (*At2g16060*: *hemoglobin 1* (*Hb1*), *At3g02550*: *LOB domain-containing protein 41* (*LBD41*)), *At1g43800*; *Acyl carrier protein* (*ACP*) *desaturase 6* (*AAD6*), *At5g15120*: *Plant cysteine oxidase 1* (*PCO1*), *At5g39890*: *PCO2*, *At4g33070*: *Pyruvate decarboxylase 1* (*PDC1*), *At2g17850*, and *At5g66985*), which are universally induced under low oxygen [31] can be found in the DEGs of *MA* and *MC-JcERFVII2* overexpressing lines (Supplementary Materials Table S1).

Since we observed more DEGs being upregulated in the *MA-JcERFVII2* overexpressing line than that of the *MC-JcERFVII2*, we carefully examined the expression of the 122 upregulated DEGs from the *MA-JcERFVII2* transgenic line (Figure 5C). Of the 122 upregulated *MA-JcERFVII2* DEGs, 22 of these were also upregulated in the *MC- JcERFVII2* overexpressing line (Supplementary Materials Table S1). The rest of them (100 genes) were not differentially expressed in the *MC-JcERFVII2* overexpressing line (Figure 5C; Supplementary Materials Table S1). A possible explanation for these results is that the increase in *JcERFVII2* protein abundance could elevate the expression of these 100 genes. GO analysis of the upregulated DEGs from the *MA-JcERFVII2* transgenic line revealed their roles in response to multiple stresses, including hypoxia (FDR: 2.60 × 10^−7^), oxidative stress (FDR: 6.60 × 10^−7^), and other organisms (1.10 × 10^−6^) (Figure 5D; Supplementary Materials Table S2).

**Figure 6:**
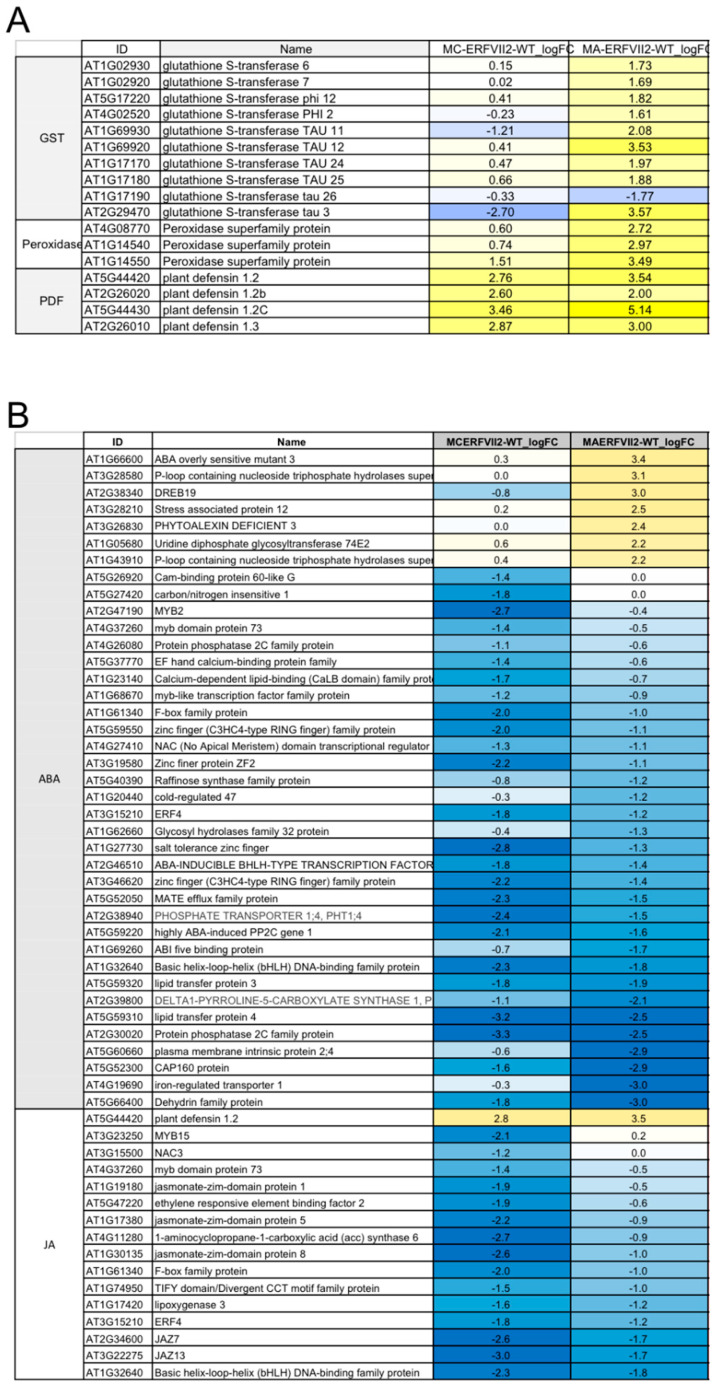
Gene expression pattern of DEGs related to (**A**) ROS scavenging and pathogen responses and (**B**) ABA and JA responses. Blue and yellow colors indicate upregulation and down regulation, respectively. Data used to generate this figure can be found in Supplementary Materials Table S1.

Based on GO enrichment results, DEGs in some specific classes demonstrated co-expression patterns (Figure 6). Several *Plant defensin* (*PDF*) genes were upregulated in both *MA-* and *MC-JcERFVII2* lines (Figure 6A, Supplementary Materials Table S1). *Glutathione transferase* and *peroxidase* genes were upregulated mainly in the *MA-JcERFVII2* line (Figure 6A, Supplementary Materials Table S1). In contrast, specific genes that function in ABA and JA responses were downregulated in both *MA-* and *MC-JcERFVII2* transgenic lines (Figure 6B; Supplementary Materials Table S1). These results altogether indicate that post-translational modification of JcERFVII2 protein under aerobic conditions can affect its regulative function.

### 2.6. Validation of JcERFVII2 Target Genes

**Figure 7:**
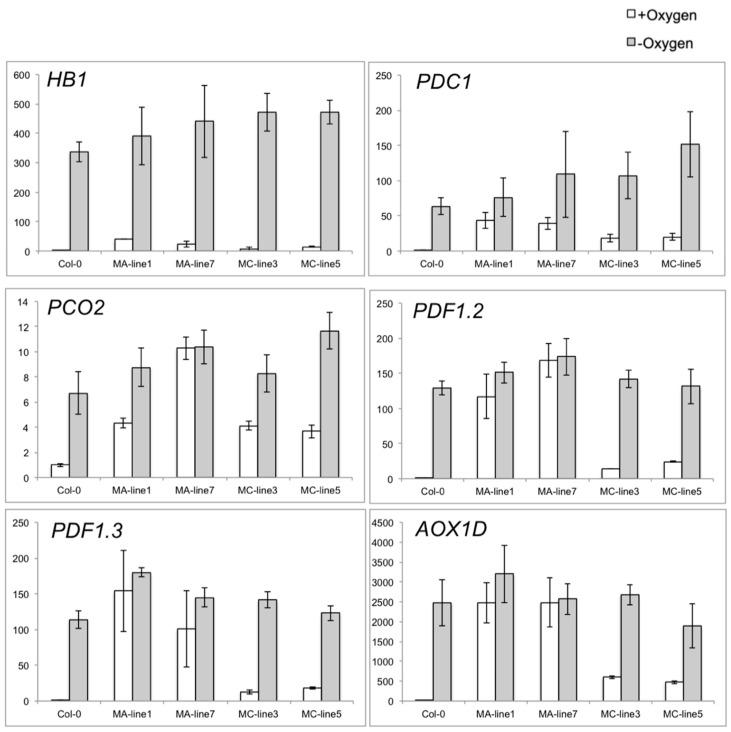
Quantitative real-time PCR validation of transcriptome data for selected genes. Relative expression was normalized to the abundance of *UBQ10*. Data represent mean ± SE (*n* = 3).

For verification of the RNA-seq results, quantitative reverse-transcription polymerase chain reaction (RT-PCR) was used to quantify 6 representative transcripts. The selected mRNAs included three core hypoxia genes (*HB1*, *PDC1*, and *PCO2*), two plant defense responsive genes (*PDF1.2* and *PDF1.3*), and *Alternative oxidase 1D* (*AOX1D*). The analysis confirmed that levels of these mRNAs are more induced in the *MA-JcERFVII2* overexpressing line than those of the *MC-JcERFVII2* and Col-0 grown in aerobic conditions (Figure 7). Furthermore, low oxygen-induced the accumulation of these mRNAs in all genotypes; however, the mRNA accumulation in some of these genes is slightly higher in the *MA* or *MC-JcERFVII2* overexpressing lines (Supplementary Materials Table S2).

## 3. Discussion

This study focuses on elucidating the roles of *JcERFVIIs* towards waterlogging and low oxygen response. Phylogenetic and domain architecture analyses reveal that *JcERFVII1* and *JcERFVII2* are orthologs of constitutively expressed Arabidopsis *ERFVII* genes, *RAP2.2* and *RAP2.12* and *RAP2.3*, respectively (Figure 1). The last member of this *JcERFVII* family, *JcERFVII3*, is an ortholog of low-oxygen induced Arabidopsis *HRE2* (Figure 1). This study reveals that the expression of *JcERFVII1* is highly constitutive and remains unaffected following waterlogging, while *JcERFVII2* and *JcERFVII3* are upregulated by waterlogging (Figure 2 and Supplementary Materials Figure S1). Analysis of *RAP2.3* in flooding tolerant Brassica species, *Rorippa sylvestris* and *Rorippa amphibia,* demonstrated that under flooding, no induction of *RAP2.3* was observed [12]. Altogether, these data indicate that *JcERFVII2* from waterlogging sensitive Jatropha and *RAP2.3* from *Brassica* plants might undergo divergent evolution in gene expression.

In the dicot model Arabidopsis, all five ERFVIIs possess conserved motif function as N-degron that promotes the degradation of ERFVIIs via oxygen- and nitric oxide (NO) dependent N-end rule pathway of targeted proteolysis [8,10,13,14]. Overexpression of all five Arabidopsis *ERFVIIs* drastically improves low oxygen tolerance by promoting the expression of the genes involved in low oxygen adaptation [13,14,16,20]. Intriguingly, overexpression of stable version of *HRE1* and *HRE2* further improved low oxygen tolerance in Arabidopsis [14], while overexpression of stable version of *RAP2.12* resulted in a reduction of plant growth in air and also decreasing submergence tolerance in Arabidopsis [13,22]. In this study, we demonstrated that the JcERFVIIs 1–3 are targeted at the N-end rule pathway in vitro (Figure 3), leading to a question of whether modulation of the JcERFVII2 stability can further improve low oxygen tolerance. Transgenic Arabidopsis lines overexpressing *MC-JcERFVII2* are highly tolerant of both flooding and low oxygen stress, suggesting that *JcERFVII2* could function as a low-oxygen determinant (Figure 4). In contrast, transgenic Arabidopsis lines overexpressing *MA-JcERFVII2* are highly sensitive to low oxygen stresses (Figure 4). Moreover, overexpression of *MA-JcERFVII2* yields a decrease in rosette size and dry-weight when grown in air (Figure 4A,B), demonstrating that modulation of the JcERFVII2 stability interferes with growth and development.

In this study, transcriptome profiling reveals that modification of JcERFVII2 stability affects transcript accumulation of multiple genes controlling cellular metabolism and stress responses (Figure 5B). Previously, Bui et al. [32] demonstrated transcriptional activity of constitutively expressed *RAP2.2*, *RAP2.3* or, *RAP2.12* on a set of hypoxia-responsive promoters. Papdi et al. [20] showed that all three *RAP2* genes, when overexpressed, can transactivate *ADH* (*alcohol dehydrogenase*) promoter. In addition, Gasch et al. [33] demonstrated that overexpression of all three *RAP2* genes induced expression of *ADH* in transgenic Arabidopsis. Similarly, our study found that overexpression of *MA-* and *MC-JcERFVII2* upregulated the expression of 8 out of 49 core hypoxia-responsive genes (Supplementary Materials Table S1). Previous studies demonstrated that ectopic expression of *ERFVIIs* in transgenic plants increased tolerance to multiple abiotic stresses [8]. Some evidence suggested that RAP2.3 functions in pathogen response and ROS detoxification. Ogawa et al. [34] showed that tobacco BRIGHT YELLOW-2 cells overexpressing Arabidopsis *RAP2.3* were more tolerant of H_2_O_2_ and heat stress. Moreover, the expression of *PDF1.2* and *GST6* was enhanced in the transgenic Arabidopsis lines overexpressing *RAP2.3* [34]. Furthermore, overexpression of the *RAP2.3* ortholog, *CaPF1* (*Capsicum annuum pathogen and freezing tolerance-related protein 1*), in Virginia pine upregulated several antioxidant enzymes including ascorbate peroxidase, glutathione reductase and superoxide dismutase [35]. In this study, we found that overexpression of both *MA-* and *MC-JcERFVII2* induced the expression of several *PDF* genes (*PDFs 1.2*, *1.2b*, *1.2C*, and *1.3*; Figure 6A; Supplementary Materials Table S1). We also observed the upregulation of several *GST* and *peroxidase* genes in the transgenic line overexpressing *MA-JcERFVII2* (Figure 6A; Supplementary Materials Table S1). Altogether, these results demonstrate that *JcERFVII2* may involve in pathogen response and reducing ROS accumulation in plant cells.

Our study demonstrated that overexpression of *MA-JcERFVII2* interferes with growth and development (Figure 4). Paul et al. [22] compared transgenic Arabidopsis lines overexpressing wildtype and stabilized forms of *RAP2.12* under aerobic conditions and found that the stabilized *RAP2.12* affected central metabolic processes by increasing activities of fermentative enzymes and accumulation of fermentative products including ethanol, lactate, alanine and γ-amino butyrate (GABA), therefore resulted in decreased ATP and starch levels. In this study, GO enrichment analysis revealed that the alpha-amino acid metabolic process was enriched in the upregulated DEGs from *MA-ERFVII2* (Figure 5D). This GO category includes genes encoding for several enzymes responsible for glutamate and GABA synthesis (*AT2G02010*: *glutamate decarboxylase 4* (*GAD4*), *AT5G37600*: *glutamine synthase* (*GSR1*); *AT5G38200*: *Class I glutamine amidotransferase-like superfamily protein*, and *AT4G35630*: *phosphoserine aminotransferase*; Supplementary Materials Table S1), implying the possibility that the transgenic Arabidopsis lines overexpressing *MA-JcERFVII2* could face carbohydrate starvation that leads to reduced growth and development.

In Arabidopsis, transcriptional activation of RAP2.12 can be counterbalanced by a trihelix transcriptional factor, namely HYPOXIA RESPONSE ATTENUATOR1 (HRA1) [36]. Giuntoli et al. [36] demonstrated that the interaction between *RAP2.12* and *HRA1* could enable an adaptive response to low oxygen, required for stress survival. Interestingly, transgenic wheat constitutively expressed the stabilized *TaERFVII.1* showed no growth defect phenotype, which resulted from the upregulation of *TaSAB18.1*, an ortholog of *HRA1*, under aerobic condition [25]. In this study, we did not observe the upregulation of *HRA1* from the transgenic Arabidopsis overexpressing both *MA-* and *MC-JcERFVII2* grown under aerobic conditions (Supplementary Materials Table S1).

Leon et al. [37] recently showed that enhanced RAP2.3 expression reduced NO-triggered transcriptome adjustment, and thus it functions as a brake for NO-triggered responses that included the activation of JA and ABA signaling in Arabidopsis. In addition, Vincente et al. [38] found that *RAP2.3* enhanced abiotic stress responses by interacting with BRM, a chromatin-remodeling ATPase, that repressed ABA responses. Gibb et al. [39] demonstrated that *RAP2.3* regulated the expression of *ABSICISIC ACID INSENSITIVE5* (*ABI5*), a major negative regulator of germination in seed endosperm. Interestingly, our study found that the NO-scavenger gene, *HB1*, was upregulated (Supplementary Materials Table S1). Additionally, genes involved in JA and ABA-activated signaling and responses were mostly down-regulated in transgenic Arabidopsis overexpressing *MA* and *MC-JcERFVII2* (Figure 6B), suggesting *JcERFVII2* could modulate NO accumulation and hormonal response.

In summary, our study demonstrated that JcERFVII2 is an N-end rule regulated waterlogging-responsive transcription factor that functions by modulating gene expression of cellular metabolic and multiple stress-responsive genes, including low-oxygen, oxidative, and pathogen response. Constitutive upregulation of fermentative and stress-responsive genes could compromise growth and development in the transgenic Arabidopsis overexpressing the stabilized *JcERFVII2*. This study highlights several possibilities for future investigation, including genetic manipulation of the *JcERFVII2* gene in Jatropha to determine whether it can improve waterlogging tolerance and elucidation of the *JcERFVII2* roles in controlling physiological responses to multiple abiotic stresses in Jatropha and other crop plants.

## 4. Materials and Methods

### 4.1. Multiple Sequence Alignment and Motif Identification

Full-length amino acid coding regions of ERFVIIs were downloaded from the Jatropha genome database (https://www.kazusa.or.jp/jatropha/) and the Arabidopsis information resource (http://www.arabidopsis.org/). Multiple sequence alignment was performed using CLUSTALW, and then a phylogenetic tree was built by the neighbor-joining method (Poisson correction, pairwise deletion of gaps) using the MEGA10 software [40]. Domain analysis was performed using MEME [41] following the models published for Arabidopsis [5].

### 4.2. Genetic Materials

*J. curcas* (cv. “Chai Nat”—a local Thai variety) and *A. thaliana* genotypes including the Col-0 accession and *35S:MC-JcERFVII2* and *35S:MA-JcERFVII2* (ectopic expression) transgenic lines were used in this study. The genome of Col-0 has already been sequenced.

### 4.3. Plant Growth and Stress Condition

*J. curcas* seedlings were grown and waterlogged, as described in Juntawong et al. [29].

For growth in soil, *A. thaliana* plants were grown in soil containing 50% (*v*/*v*) peat, 25% (*v*/*v*) perlite, and 25% (*v*/*v*) coconut fiber with regular irrigation in a growth room at 120 μmol photon m^−2^ s^−1^ 16 h light/8 h dark, at 23 °C. Submergence stress was performed using 10 leaf-stage plants grown in 5-cm^2^ pots by placing them in a plastic container completely filled with water for 3 d.

For growth in sterile culture, *A. thaliana* seeds were surface sterilized, stratified at 4 °C for 48 h and plated on 0.5× solid Murashige and Skoog (MS) medium (0.215% (*w*/*v*) MS salts containing 1% (*w*/*v*) agar, pH 5.7) in 20-mm^2^ dishes. Growth was in a vertical orientation in a growth room. Hypoxia stress was performed under dim light at the end of the 16-h light cycle in a sealed argon chamber. For hypoxia stress, 98% argon and 2% oxygen mixture was passed through water and into the chamber while ambient air was pushed out by positive pressure. Control was placed in an open chamber side by side.

### 4.4. Quantitative Reverse Transcription PCR

Total RNA samples were extracted using TRIzol reagents (Thermo Fisher Scientific, Waltham, MA, USA), subjected to DNase treatment, and RNA cleanup using an RNA-mini kit (Qiagen, Hilden, Germany). Three replicates of total RNA samples were used. One microgram of total RNAs was used to construct cDNA using MMuLv reverse transcriptase (Biotechrabbit, Berlin, Germany) in a final volume of 20 μL. The cDNA was diluted five times. Quantitative-realtime PCR (qPCR) reaction was performed according to Butsayawarapat et al. [42] using QPCR Green Master Mix (Biotechrabbit, Berlin, Germany) on a MasterCycler RealPlex4 (Eppendorf, Hamburg, Germany). For each sample, the PCR reaction was performed in triplicate. Each reaction contained 1 μL of diluted cDNA, 0.5 μM of each primer and 10 μL of QPCR Green Master Mix in a final volume of 20 μL. The PCR cycle was 95 °C for 2 min, followed by 45 cycles of 95 °C for 15 s and 60 °C for 30 s. Amplification specificity was validated by melt-curve analysis at the end of each PCR experiment. Relative gene expression was calculated using the 2^−∆∆CT^ method. Primers used to study Jatropha’s gene expression were previously reported by Juntawong et al. [29]. The genes and primers used in Arabidopsis are shown in Supplementary Materials Table S3.

### 4.5. Analysis of Protein Stability

To construct the plasmids used for in vitro protein stability assay, cDNAs were amplified from *J. curcas* total cDNA using gene-specific primers (Supplementary Materials Table S3). The PCR products were ligated into a modified version of the pTNT (Invitrogen, Carlsbad, CA, USA) expression vector (pTNT-3xHA) [14]. N-terminal mutations were incorporated by modifying the forward primer sequences accordingly (Supplementary Materials Table S3).

For in vitro protein expression, TNT T7 Coupled Reticulocyte Lysate System (Promega, Madison, WI, USA) and 2 µg plasmid template was used according to manufacturer’s guidelines. Where appropriate, 100 mM MG132 (Sigma, St. Louis, MO, USA) was added. Reactions were incubated at 30 °C. Samples were taken at indicated time points before mixing with protein loading dye to terminate the reaction. Equal amounts of each reaction were subjected to anti-HA immunoblot analysis. All blots were checked for equal loading by Coomassie Brilliant Blue staining.

For immunoblotting, proteins resolved by SDS-PAGE were transferred to PVDF using a MiniTrans-Blot electrophoretic transfer cell (Bio-Rad, Hercules, CA, USA). Membranes were probed with HA-probe (Y-11) HRP (Santa Cruz, CA, USA) at a titer of 1:1000. Immunoblots were detected using TMB (Tetramethyl Benzidine; Thermo Fisher Scientific, Waltham, MA, USA) solution (Invitrogen, Carlsbad, CA, USA).

### 4.6. Generation of Transgenic Lines

To construct Ti binary plasmids for plant transformation, *JcERFVII2* open reading frame was amplified by RT-PCR from RNA extracted from roots of *J. curcas* using gene-specific primers (Supplementary Materials Table S3). The PCR product was inserted into the pCXSN binary plasmid [43], transformed into *E. coli* DH5α, and selected with 50 μg mL^−1^ kanamycin. N-terminal mutations were incorporated by changing the forward primer sequences accordingly (Supplementary Materials Table S3). The pCXSN, a plant overexpression vector, provides a CaMV 35S promoter and nopaline synthase terminator sequence in a Ti binary plasmid with a hygromycin-resistant gene. After sequence confirmation, the plasmid was electroporated into *Agrobacterium tumefaciens* GV3101 and colonies selected with 50 μg mL^−1^ kanamycin. Col-0 transformation was performed according to Clough and Bent [44]). T1 seeds were collected, seedlings resistant to 35 μg mL^−1^ hygromycin were propagated, and homozygous single insertion events were established.

### 4.7. RNA-Seq, Differential Gene Expression Analysis, and Gene Ontology Enrichment

Total RNA samples were extracted using TRIzol reagents (Thermo Fisher Scientific, Waltham, MA, USA), subjected to DNase treatment, and RNA cleanup using an RNA-mini kit (Qiagen, Hilden, Germany). Two replicates of total RNA samples were used for transcriptome analysis according to the ENCODE recommended RNA-seq standards (https://genome.ucsc.edu/ENCODE/protocols/dataStandards/ENCODE_RNAseq_Standards_V1.0.pdf). The integrity of the RNA samples (RIN) was evaluated on an RNA 6000 Nano LapChiprun on Agilent2100 Bioanalyzer (Agilent Technologies, Waldbronn, Germany). Samples with a RIN > 7 were used in RNA-seq library preparation.

For each sample, 3 μg of total RNAs were used to generate a sequencing library using an Illumina^®^ TruSeq^TM^ RNA Sample Preparation Kit v2 (Illumina, San Diego, CA, USA). Paired-end, 100 bp RNA-seq was performed on a NovaSeq6000 platform. FASTQ files were generated with the base caller provided by the instrument. Quality control filtering and 3′ end trimming were analyzed using the FASTX-toolkit (http://hannonlab.cshl.edu/fastx_toolkit/index.html) and Trimmomatic software [45], respectively. The raw read files were deposited in the NCBI GEO database under the accession numbers GSE154601.

Differential gene expression analysis was performed according to Juntawong et al. [29]. The FASTQ files were aligned to the reference transcriptome using TopHat2 software (v2.0.13) [46]. A binary format of sequence alignment files (BAM) was generated and used to create read count tables by the HTseq python library (citation). Differentially-expressed genes were calculated using the edgeR program [47] with an FDR cutoff of <0.05.

Gene ontology (GO) enrichment was analyzed using AgriGO V2.0 [48]. For visualization, REViGO was applied to summarize and removing redundant GO terms [49].

## Supplementary Materials

The following are available online at https://www.mdpi.com/2223-7747/9/9/1068/s1, Data S1: Nucleotide and amino acid sequences of JcERFVIIs, Figure S1: Gene expression level of *JcERFVIIs 1*, *2*, and *3* retrieved from the JCDB database, Figure S2: Expression analysis of *JcERFVII-2* and five members of the Arabidopsis *ERFVIIs* in transgenic lines. (A) semi quantitative RT-PCR analysis of *JcERFVII-2* in the transgenic Arabidopsis plants overexpressing *MA*- or *MC-JcERFVII-2.* Actin was used as a control. (B) Means of Count Per Million (CPM) gene expression values of the Arabidopsis *ERFVII* genes obtained from RNA-seq experiment, Table S1: Differentially expressed genes, Table S2: Gene ontology enrichment results. Table S3: Primers used in this study.
