# Supplementary material for: Overexpression of Jatropha curcas ERFVII2 Transcription Factor Confers Low Oxygen Tolerance in Transgenic Arabidopsis by Modulating Expression of Metabolic Enzymes and Multiple Stress-Responsive Genes
Source: Plants (Basel). 2020 Aug 20;9(9):1068. doi: 10.3390/plants9091068 (PMC7570394; doi:10.3390/plants9091068)
Supplement: Supplementary file 1 [file plants-09-01068-s001.zip › plants-887819-supplementary-re2/Data S1.pdf]

>JcERFVII1
ATGTGTGGCGGTGCTATCATCTCCGACTTTATACCTCCCGCGGTCGCTGGGCGATCTTCTCGGCGGTTGACAGCTGATTT
TCTTTGGCCTGATCTAAAGAAACCCATTGGAAAGCAGTACTCAAAACCTGTTGTTGATCTTGACAATGATTTCGAGGCTG
ATTTTCAGGAGTTCAAAGATGAATCTGATGCCGATGAGGAAGATGATGTCATGCTTGATGTCAAGCCTTTTGCTTTTTCT
GCTACTGCCTCTCCTCCTGCTCGCAATCGCTCTTCCTCTCGTGGATCTGCAGCTGTCAAATCTGTGGAATTCAATGGGCA
GGCAGAAAAATCTGCAAAAAGAAAGAGAAAGAACCAGTATAGAGGAATCCGGCAGCGCCCATGGGGAAAATGGGCTGCT
GAGATTCGTGATCCCCGGAAAGGGGTGCGTGTCTGGCTAGGAACATTCAATACTGCAGAAGAAGCTGCAAGAGCATATGA
CGCGGAGGCACGTAGAATTCGTGGCAAGAAAGCTAAAGTTAACTTCCCCGAGGAAGCTCCGCATGCTTCACCAAAACGTC
CATCTAAGGCAAACTCTCAGAAATCACTTGGCAAAACAAACTTGGCTGAGAATTTGAATTACTTGGACAATCCAGAACAGG
ACTACTTCAATTCTATGGGCTTTGTTGAAGAGAAACCGCCAGTGAGCCAGTTTGGTATAATGGACTCTCTTCCTGTTAAT
GGAGATGCTGTAGTAAACTCCATTACTCCAAGTGACAATGTTCCCATGTATTTCAATTCTGATCAAGGGAGCAACTCTTT
TGAGTGTTCTGACATTGGATGGGCAGAGCAGGCTGCAAAGACTCCTGAAATCTCATCTGTTCTCTCAGCTACTCCTGAAA
TTGACGAATCTCTATTTATAGAGGATGCTAACCCCAAAAAGAAGCTGAAATCTGACTCTGGCAATGAGGCGCCTGCAGAA
GAAAACAATGGAAAATCTCTGTCTGATGAGCTGTTGGCCTTTGAAAACCAGATGAACTATCAGATGCCTTATCTCGAGGG
GAGTTGGGAAGCTTCGCTTGATAGCTTCCTGAATGGAGACACAACTCAGGATGGCGGAAACCCAATGGACCTGTGGAGCT
TTGATGACCTCGCTGGCATGGTTGGGGGTGTTTATTGA

>JcERFVII2
ATGTGCGGAGGTGCTATTATTTCAGATTTCGTCGCCGTGAAACGTAGCCGGCGATTGAACGCCGAGGATCTCTGGTCTGA
GCTTGACACTTTCTCTGACTTTCTTGGCTTGGATTATCCTAATAATGGCAATAAAGAAGTCCTGCCTTCCCACTTTGATCTTG
AACTCCCTCAAAAGCCGAACCAGCTCAACGAAGTGGAGAAGATAACAGAGAAGGCAAGTCATAGTCAAGGCGATGTAAAG
GAAGGGAACAAGACCAAGAGAACTCGAAAGAATATTTACAGGGGAATAAGGCGAAGGCCATGGGGAAAATGGGCGGCTG
AAATCAGAGACCCGCACAAGGGCGTACGTGTTTGGCTTGGTACTTACAACACAGCTGAAGAAGCCGCTCGAGCCTACGAT
GAAGCTGCCAAGCGTATCCGTGGTGAAAAAGCCAAGCTTAATTTTGGCCAGTCACCCCCATTAGCCCCAGCTCTGGTACC
TCCATACAAGAAGCGTTGCTTGATCAATCCTGGCATGGCCACTGCTAGCTACCAAATGACCGCGCCACCGCCAGAACCGT
ACGCGAGTCTCGGTTACCAAAAAGAAATGGCTAGTGATTATAAGCTGAAAGAGCAAATATCGAGCCTGGAAATGTTTCTCG
GTTTGGAGCCTGAAGAGATGGCAGCTCAGCTGAGTTGTGAGGGGTTTAACTCGGTGGACCTATGGATGCTGGGTGACCTC
GTCAGCCACCATCAGAATCATGGCCTACTCCCCTATTAA

>JcERFVII3
ATGTGTGGCGGTGCTATTATAGCTGATCTCATCCCTCGCAACCGTGGCCGTGGCGTCTCCTCTTCTGACCTCTGGCCTGA
ATCCGCCTTCTCTAAACTCAATCCCTTCCAGTCTTATCCGAGCTCATTCGGCAACCATGATTCGTTCACTCTTAAAAGATCC
CAACCTGCTTCAGGTGATAGGCAAGTAGAGAAGACACCTAAAAAAAGGCAAAGGAAGAACTTGTACAGAGGTATAAGACAA
CGACCCTGGGGCAAATGGGCAGCTGAAATTCGTGATCCAAGAAAGGGAGTCCGTGTTTGGCTTGGGACTTTCAACACTGC
CGAAGAAGCTGCGAGAGCTTACGATAGAGAAGCGCGTAAAATTCGAGGCAAGAAAGCCAAAGTCAATTTCCCTAATGAGG
ATGATTCCTACACCATTTCTCAAAATCACCTTAACGGACATAATCATAGAAACAATCCTCCTTTTTATCAGCCTCTCACTTGT
AATTTCAACAACACTCCCAAAAGTTATGATTTTGGGTTCGGTTACGATTTAAACCATATTGGATCTTATAATTCAAATGGTAT
GAACACCGAACCTGTTATCGTTTCTGGCGAAGAGATTTCTGGGTCTGGTTCAGAGGAGGAAGCCTACTCACTGATAGGCA
ATAATTACATGGGCGAAGTAAAAGTTAAACAGGAAGAAGAGAAAATGGAAGTGGAGAAAGTGAAAAACAGAGAGGTAGTGA
TAGTGGACTTAGAATCAGAGCAGCAAGAGAGCGAGCTGCAGAAATTATCAGAGGAACTAATGGCTTATGAGAATTACATGA
AATTTTATCAGATTCCTTATCTGGATGGGCAGTCAACGGCACCGAATACGGCTCCTCAGGAAAGCGTTGTTGGAAATCTCT
GGAACTTCGACGATGATAGTGTTGCTGCTCCGGTAACGTCTTCTGCTCTCTAA

>JcERFVII1
MCGGAIISDFIPPAVAGRSSRRLTADFLWPDLKKPIGKQYSKPVVDLDNDFEADFQEFKDESDADEEDDVMLDVKPFAFSATASP
PARNRSSSRGSAAVKSVEFNGQAEKSAKRKRKNQYRGIRQRPWGKWAAEIRDPRKGVRVWLGTFNTAEEAARAYDAEARRIR
GKKAKVNFPEEAPHASPKRPSKANSQKSLGKTNLAENLNYLDNPEQDYFNSMGFVEEKPPVSQFGIMDSLPVNGDAVVNSITP
SDNVPMYFNSDQGSNSFECSDIGWAEQAAKTPEISSVLSATPEIDESLFIEDANPKKKLKSDSGNEAPAEENNGKSLSDELLAFE
NQMNYQMPYLEGSWEASLDSFLNGDTTQDGGNPMDLWSFDDLAGMVGGVY

>JcERFVII2
MCGGAIISDFVAVKRSRRLNAEDLWSELDTFSDFLGLDYPNNGNKEVLPSHFDLELPQKPNQLNEVEKITEKASHSQGDVKEGN
KTKRTRKNIYRGIRRRPWGKWAAEIRDPHKGVRVWLGTYNTAEEAARAYDEAAKRIRGEKAKLNFGQSPPLAPALVPPYKKRCL
INPGMATASYQMTAPPPEPYASLGYQKEMASDYKLKEQISSLEMFLGLEPEEMAAQLSCEGFNSVDLWMLGDLVSHHQNHGLL
PY

>JcERFVII3
MCGGAIIADLIPRNRGRGVSSSDLWPESAFSKLNPFQSYPSSFGNHDSFTLKRSQPASGDRQVEKTPKKRQRKNLYRGIRQRP
WGKWAAEIRDPRKGVRVWLGTFNTAEEAARAYDREARKIRGKKAKVNFPNEDDSYTISQNHLNGHNHRNNPPFYQPLTCNFN
NTPKSYDFGFGYDLNHIGSYNSNGMNTEPVIVSGEEISGSGSEEEAYSLIGNNYMGEVKVKQEEEKMEVEKVKNREVVIVDLES
EQQESELQKLSEELMAYENYMKFYQIPYLDGQSTAPNTAPQESVVGNLWNFDDDSVAAPVTSSAL
