# Supplementary figures and images for: Overexpression of Jatropha curcas ERFVII2 Transcription Factor Confers Low Oxygen Tolerance in Transgenic Arabidopsis by Modulating Expression of Metabolic Enzymes and Multiple Stress-Responsive Genes
Source: Plants (Basel). 2020 Aug 20;9(9):1068. doi: 10.3390/plants9091068 (PMC7570394; doi:10.3390/plants9091068)

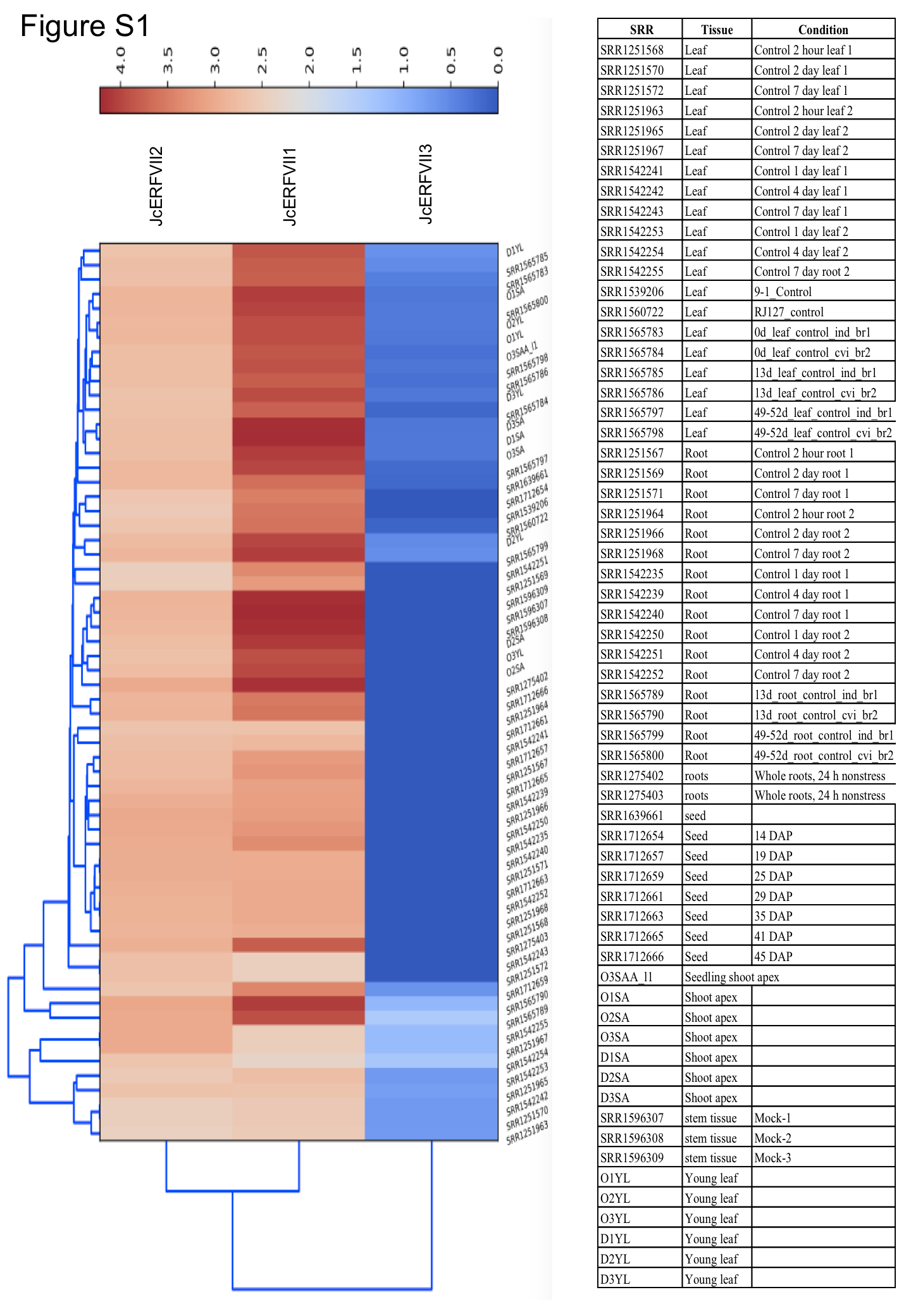

Supplement: Supplementary file 1 [file plants-09-01068-s001.zip › plants-887819-supplementary-re2/FigureS1.tiff]

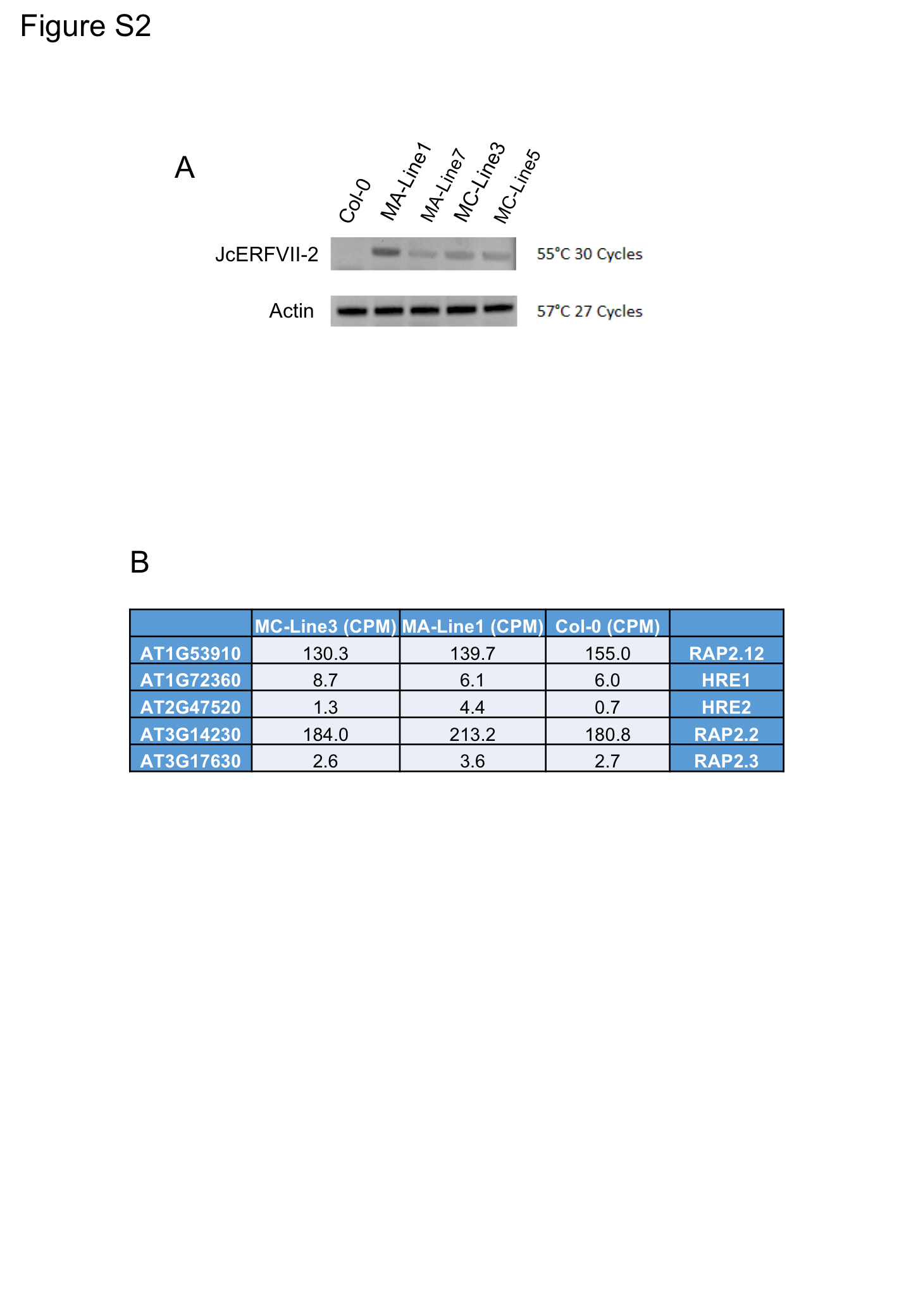

Supplement: Supplementary file 1 [file plants-09-01068-s001.zip › plants-887819-supplementary-re2/FigureS2.tiff]
